# Effects of dietary supplementation of myco-fabricated zinc oxide nanoparticles on performance, histological changes, and tissues Zn concentration in broiler chicks

**DOI:** 10.1038/s41598-022-22836-3

**Published:** 2022-11-05

**Authors:** M. H. Hatab, E. Rashad, Hisham M. Saleh, El-Sayed R. El-Sayed, A. M. Abu Taleb

**Affiliations:** 1 Biological Application Department, Nuclear Research Center, Egyptian Atomic Energy Authority, Cairo, Egypt; 2 Cytology and Histology Department, Cairo University, Giza, Egypt; 3 Plant Research Department, Nuclear Research Center, Egyptian Atomic Energy Authority, Cairo, Egypt

**Keywords:** Biochemistry, Biotechnology, Physiology, Zoology

## Abstract

A five weeks biological experiment was planned to investigate the impacts of dietary supplementation with zinc oxide nanoparticles (ZnONPs) synthesized by the endophytic fungus *Alternaria tenuissima* on productive performance, carcass traits, organ relative weights, serum biochemical parameters, histological alteration in some internal organs and concentration of this element in the serum, liver, thigh and breast muscle in broiler chicks. A total of 108 3-day-old commercial broiler chicks (Cobb 500) were individually weighed and equally distributed in a completely randomized design arrangement according to the dose of ZnONPs supplementation into 3 dietary experimental groups. There were 6 replications having 6 birds per replicate (*n* = 36/ treatment) for each treatment. The three experiential dietary treatments received corn-soybean meal-based diets enhanced with 0 (control), 40 and 60 mg/kg diet of ZnONPs respectively with feed and water were provided ad libitum consumption through 5 weeks life span. Present results indicated that after 5 weeks of feeding trial and as compared to control, the ZnONPs supplementation groups recorded higher body weight, improved feed consumption, feed conversion ratio and performance index. Serum biochemical analyses revealed that serum cholesterol**,** triglyceride, low density lipoprotein and uric acid decreased significantly, while high density lipoprotein and liver enzyme concentrations were increased significantly. Meanwhile, zinc accumulation in serum, liver and breast and thigh muscle were linearly increased with increasing zinc supplementation. It could be concluded that supplementation of ZnONPs to broiler diet at 40 or 60 mg/kg improved productive performance, birds' physiological status and the lower levels Zn (40 mg/kg diet) revealed promising results and can be used as an effective feed additive in broilers.

## Introduction

Poultry industry is an important economical business and fastest growing sector in the agriculture field, which holds the primacy in meeting the needs of the population in meat and eggs and its processed products that increase the nutritional quality of human food. It has become an attractive industry because of its rapid outcomes^1^. Poultry diets that meet the requirements with availability of minerals and high-quality is the most important factor to ensure high animal productivity. The animals’ need for minerals can be met through various forms of compounds: inorganic, organic, chelated and nanoscale.

Zinc (Zn) among the trace minerals had widely varying functions and biological activities in several physiological, digestive, and metabolic processes in birds^2^. Zn is a cofactor of several enzymes essential for metabolism and reproduction, appetite control, growth, and glandular development^3^. Furthermore, Zn plays a crucial role in secretion of important hormones secretion such as growth hormones^4^, insulin hormones, nucleic acid synthesis, gene expression, cellular division, signal transduction, gene transcription, protein synthesis^5^. Besides, it is a vital free radical’s scavenger of the antioxidant defense system^6^.

The National Research Council recommended level of Zn 40 mg/kg in broiler diets, on the other hand, with continued genetic modification of commercial broiler strains, this level is no longer provided sufficient growth, health and production for modern broiler strains^7^. Consequently, poultry producers added these salts to the diets at levels higher than recommended by National Research Council to meet bird requirements, which results in higher production costs and increases mineral excretion in feces causing environmental pollution especially at the large scale of breeding^8^. In addition, higher inclusion levels of Zn may influence other microelements’ balance, decrease the stability of vitamins and other nutrients, and increase its accumulation inside the animal body^6^.

Traditionally Zn is usually supplemented in the poultry feed in the inorganic forms such as sulfates, oxides and chloride due to its lower cost and commercial availability. However, there may be interactions and antagonism among trace minerals, preventing their absorption in body thus impair Zn absorption due to the pH changes that naturally occur in the digestive tract of poultry. Many authors documented that the inorganic trace minerals may be replaced by organic sources to reduce the over supplementation and decrease their excretion^9–11^. The supplementation of organic Zn in broiler diets is limited until now due to its relatively high cost^6^. Therefore, improving the bioavailability of Zn may help to solve these problems.

The use of Nanoparticles of mineral elements, represents an emerging and promising approach in the animal farming industry. Minerals in the form of nano particles showed higher bioavailability^12^, due to their novel characteristics, including high surface area and activity, surface active centers, high catalytic efficiency and stronger adsorbing ability as well as it can be easily carried up by the gastrointestinal tract and further distribute into the blood and utilized in the animal system and reached too deeper tissues by more efficient than the larger sized particles^13^.

Nanoparticles of zinc oxide are considered as a promising alternative feed supplement for poultry with a claim of exerting a superior efficacy compared with both inorganic and organic forms^14^. Previous reports indicated that Nano-zinc outweighed the conventional Zn sources, in terms of productive and reproductive performance, carcass traits, bone development, antioxidant defense and act as high antibacterial agent, antifungal, and growth promoter ability, improving the GIT microbial population (Zhao et al. 2014), cell repair and enhancing the immune system^6,15,16^.

Currently, biogenic nanoparticles are gaining great importance for their applications in several medical and industrial sectors^17,18^. In particular, the production of NPs using microorganisms provided several advantages over other techniques and is increasingly being explored^19–21^. Besides, nanoparticles synthesized by green technologies not physical or chemical processes involving hazardous chemicals or substances which may reduce the adverse effects on digestion and absorption of zinc. This makes the use of zinc produced by the green method safe at the dose suggested in this study.

In this regard, the microbial biosynthesis of ZnONPs (by *Alternaria tenuissima*) and their physical characterization as well as their biological activities have been previously demonstrated^22^. As a part of the continuing research work in this concern, the present work was designed to valorize the effect of supplementation of two levels of ZnONPs in diets on growth performance, carcass traits, serum biochemical profile, histological alteration, and tissue concentrations of Zn in broiler chicks.

## Material and methods

### Birds’ accommodation, husbandry, diets and experimental design.

All methods used in this study are reported in accordance with ARRIVE guidelines. The Animal care and experimental protocol were reviewed and approved in accordance with guidelinesof the local ethics committee for care and use of animal in a humane manner under the approval protocol number 212/19/1/2022 by the institutional council of Atomic Energy Authority, Egypt.

A total number of one hundred and twenty-day old newly hatched broiler chicks (unsexed males and females, Cobb) were obtained from a local hatchery were housed in batteries and fed recommended diets without any treatment till the third day of age as the adaptation and quarantine period to confirm that they were free of pathogen and any other disease.

At the third day of chicks age (experimental initiation), all chicks were weighed individually and the upper and lower body weights were withdrawn. The remainder 108 chicks were used in this study from 3 day to the end of experiment at 5 weeks of age (experimental termination). The chicks were weighed individually (initial weight) and randomly distributed equally in cages in a completely randomized design into 3 different dietary treatment groups (similar in average body weight) according to the dose of zinc oxide nanoparticles (ZnONPs) supplementation. Each treatment consisted of 36 chicks which further subdivided into 6 replicates (cages) having 6 chicks in each. All replicates were separately housed by positioning at random in cages batteries at the same room. The experimental dietary treatments included the basal diet (T1 control), supplemented with 40 mg ZnONPs/kg diet (T2) and 60 mg/kg diet of ZnONPs (T3). Chicks were housed and reared in wire-mesh floor stainless steel clean galvanized batteries cages in an environmentally controlled open–sided house under the same uniform management, standard hygienic and environmental conditions. The batteries were provided by suitable feeder and fresh water via stainless steel drip nipple drinkers, which were adjusted continuously with the age of the birds, provide sufficient feeding and watering space for the birds and allowing the birds to be unrestricted (ad libitum) access to feed and water during the experimental period. Chicks received a continue lighting program of 24 h light/day throughout the first week and 23 h light: 1 h dark cycle per day until the experiment termination. House temperature was adjusted using electric heaters and maintained at 34 °C during the first 3 d of life and then was reduced gradually 2–3 °C weekly until reaching 24 °C at the end of 5th week and the vaccination program was carried out during the experiment as per schedule.

The experimental diets were formulated and varied according to feeding stage and in between groups in ZnONPs levels. Birds were fed according to 3-phase feeding period; for starter period (0–12 day of age; contained 22.14% CP and 2811 kcal/ME kg diet), and subsequently grower (13–28 day; contained 20.42% CP and 2955 kcal/ME kg diet) and finisher (28–35 day; contained 18.2% CP and 3088 kcal/ME kg diet).

All experimental diets were fed in mash form and the nutrient content of the diets was calculated based on the chemical composition of the feedstuffs to meet or exceed the feeding standards of the National Research Council^7^ requirements for broilers except for Zn. The experimental diet composition and chemical analysis are presented in Table 1. Starter, grower and finisher diets contained 25.88, 24.62, and 23.12 mg/kg of Zn from raw materials, respectively. The estimated Zn requirements in the current experiment were higher than those (40 mg/kg of diet) rec-ommended by the National Research Council (NRC1994). Nevertheless, they were lower than those (added 100 mg/kg of diet Zn) recommended Cobb-500 manuals (Cobb–Vantress 2021).

**Table 1:** Ingredient composition and calculated chemical analysis of the basal diets.

Ingredient composition (kg)	Starter	Grower	Finisher
Yellow corn	56	59	64
Soybean meal 44%	39.5	35	29
Vitamin and mineral premix	0.3	0.3	0.3
Monocalcium phospate	1.5	1.4	1.3
Sodium chloride	0.2	0.2	0.2
Bicarbonate	0.2	0.2	0.2
Vegetable oil	0.6	2.2	3.3
limestone	1.1	1.1	1.1
DL-methoinin	0.3	0.3	0.3
L-lysin	0.2	0.16	0.18
Cholin chloride	0.15	0.15	0.15
Calculated chemical analysis			
Crude protein%	22.14	20.42	18.2
ME (kcal/kg)	2811	2955	3088
Calcium%	0.95	0.82	0.8
Lysine%	1.4	1.29	1.15
Nonphytate *P*%	0.5	0.47	0.45
Methionine%	0.65	0.63	0.6
Zn (mg)	25.88	24.62	23.12

Vitamin-mineral premix provided per kg of diet: vitamin A, 14,000 IU; vitamin D3, 3,000 IU; vitamin E, 34.5 IU; vitamin K3, 4 mg; biotin, 0.15 mg ;vitamin B1, 1.3 mg; vitamin B2, 6 mg; pantothenic acid, 20 mg; vitamin B6,3 mg; niacin, 60 mg; vitamin B12, 6 mg; choline, 150 mg; folic acid,0.5 mg. Mn, 60 mg; Fe, 50 mg; Cu, 10 mg; I, .1.5 mg; Se, 0.2 mg; Zn, 0 mg.

### Zinc oxide nanoparticles (ZnONPs) synthesis, preparation and purification.

ZnONPs were synthsized according to the method described in details^22^ using the cell free culture filtrate of the fungus *Alternaria tenuissima* AUMC10624 (Culture Collection of Assiut University Mycological Center, Assiut, Egypt, http://www.aun.edu.eg/aumc/aumc.htm). Fungal spores from the cultures (7 days old) of *A. tenuissima* were harvested and the spore concentration was adjusted to a concentration of 10^6^ spores/ml. Spore suspension was transferred in vial sealed with paraffin and irradiated by gamma rays at a dose of 500 Gy, the best irradiation dose for maximum synthesis of ZnONPs^23^. Irradiation process was carried out at the Nuclear Research Center (Cairo, Egypt) using ^60^Co Gamma chamber, MC20, Russia, with an average dose rate of 605.726 Gy h^−1^ at the time of the experiment.

After irradiation, 1 mL of the irradiated spore suspension was added to 250 mL flasks containing 50 mL medium. The flasks were incubated for 10 days at 30 °C and the ATCF was used for the production of ZnONPs.

The synthesized ZnONPs were characterized according to the method described in details by^22^. ZnONPs had a single-phase crystalline structure, according to the XRD analysis. Dynamic light scattering analysis revealed the mono-dispersion of ZnONPs and the recorded polydispersity index value was 0.308. Zeta potential value of − 23.81 mV confirmed the high stability of ZnONPs. Transmission electron microscope revealed the spherical shape and the mean particle size was 15.51 nm.

### Sampling and measurements

#### Productive performance

Production parameters were evaluated for each replicate in terms of live body weight (LBW), feed consumption (FC) feed conversion ratio (FCR), mortality and performance index (PI). Broiler chicks were weighed individually to the nearest gram at the beginning of the experiment and then weekly intervals until the end of the experiment to evaluate weekly LBW. Mortality was observed and recorded daily. Feed consumption was recorded weekly and adjusted for mortalities until the end of the experiment. Adjusted FCR was calculated at the end of experiment for each group as gm feed/gm body weight, considering the weight of dead birds^24^

#### Carcass characteristics, organs index and blood biochemical analysis

At the end of experimental period, 12 birds of each group (2 birds from each replicate) were chosen based on average body weight in each cage for both blood sampling analysis and removed digestive organs to determine carcass characteristics. Chicks were deprived from feed overnight with free access to water to decrease the direct effect of feed on blood parameters before slaughtering. After feed deprivation, birds weighed individually, slaughtering, defeathering and carcass weight was determined after removal of feathers, feet, head, and internal organs. The eviscerated carcass yield, and internal organs (liver, kidneys, gizzard, proventriculus, heart, and intestine) were extracted, blotted to dry, weighed individually and calculated as a percentage of fasted live body weight. At the same time, liver, kidney and intestine were used for histological study.

During bleeding, individual blood samples were collected from birds into a sterile dry clean centrifuged tube without anticoagulant, and then kept at room temperature for one hour to allow clotting of blood. After which the clot was rotated gently away from the tube wall using a clean fine metal wire, Separation of serum was carried out by centrifugation of coagulated blood at 3000 rpm for 10 min. The clear serum was carefully harvested and transferred to dry sterile screw capped tubes (Eppindrof tube) and stored at − 20 °C for subsequent chemical analyses.

serum constituent of glucose and cholesterol (chol), triglyceride (TG), high-density lipoprotein (HDL), low-density lipoprotein(LDL) as a lipid profile assay and aspartate amino transferase (AST), alanine amino transferase (ALT) as a liver function and uric acid and creatinine (creat) as a kidney functions were calorimetrically analyzed using commercial analytical kits (Bio-diagnostics) according to the procedures outlined by the manufacturers and were determined by SHIMADZU UV 1601 spectrophotometer.

#### Estimation of tissue and serum Zn metal concentrations (ppm)

Tissues and serum Zn concentrations were determined by inductively coupled plasma optical emission spectrometer (Leeman Prodigy High Dispersion ICP-OES, USA. The registered values for zinc were expressed as (ppm).

The technique to obtain zinc concentrations in the liver, breast, and thigh samples was adapted from previous report^25^. A total of 10 samples of each tissue (liver, breast and thigh muscle) from each bird were collected from 12 birds (2/replicate) per each group. The sample were oven dried at 100 °C for 24 h and finely ground for mineral analysis, Briefly, 0.5 g of ground samples digested with 5 mL of concentrated HNO_3_ and 2 ml of H_2_O_2_ in a 50-mL calibrated flask at 120 °C under a heating plate until the solution became clear. The content was further digested using 5 mL of concentrated HNO_3_ under a heating plate at 100 °C. A blank and the digested samples were then allowed to cool and filtered through Whatman 42 filter paper. The 1 ml extract was then diluted using deionized water to the required volume. To determine serum Zn concentration, the procedure was the same as that of the tissues analysis but with using 1 ml of serum sample.

#### Histological examination

Liver, kidney and intestine from the same 12 birds per group (2 birds/replicate) slaughtered were selected for histological studies to evaluate the presence of histopathological lesions. The procedure for histopathological preparation was applied as explained by Bancroft and Stevens^26^. Briefly, tissues were silced to 3–4 mm thick, fixed in 10% neutral buffered formalin, dehydrated in graded concentrations of ethanol, cleared in xylene and embedded in paraffin. The paraffin blocks were sectioned with a microtome at (4–6 μm) thickness and dyed with Hematoxylin and Eosin stain to study general tisuue structures. Stained sections by Hematoxylin and Eosin stain were examined using Leica microscope (CH9435 Hee56rbrugg) (Leica Microsystem, Swithereland).

### Statistical analysis

Observed data were subjected to statistically analyzed by one-way analysis of variance (ANOVA) as a completely randomized design using the general linear models (GLM) procedure under statistical analysis system software. Significant differences among the means were determined by using Duncan’s multiple-range test at *P* < 0.05. The data are presented as means ± standard error.

### Ethics approval and consent to participate

All methods used in this study are reported in accordance with ARRIVE guidelines. The Animal care and experimental protocol were reviewed and approved in accordance with guidelinesof the local ethics committee for care and use of animal in a humane manner under the approval protocol number 212/19/1/2022 by the institutional council of Atomic Energy Authority, Egypt.

## Results

### Productive performance

Generally, no mortality was observed and the general health status of broilers in all groups was good throughout the experimental period. The overall growth performance results are shown in Table 2, revealed beneficial effects for supplementation ZnONPs to broiler diets. Table 2 summarized the effect of dietary ZnONPs supplementation at two experimental levels on body weight of broiler chicks. It could be noticed that the birds had approximately the same initial body weight at the beginning of the experiment in all dietary treatments. During the whole experimental weeks, the chicks fed on dietary ZnONPs supplementation at both levels had the heaviest live body weight, in comparison with those fed on control diet, with notice that T2 was the highest value followed by (T3) as compared with the T1 (control) at all experimental weeks.

**Table 2:** Effect of ZnONPs supplementation on total body weight (g), feed consumption (FC), cumulative feed conversion ratio (FCR) and performance index (PI) of broiler chicks.

Groups	Total weight	Weekly feed consumption (FC) (g/chick)					Cumulative FC (kg)	Cumulative FCR
		Week 1	Week 2	Week 3	Week 4	Week 5		
T1	2004.26	255	334.57	670.30	890.44	1106.2	3.257	1.625
T2	2301.65	260.74	528.70	738.57	903.43	1105.5	3.537	1.537
T3	2189.17	241.48	504.65	673.44	924.30	1093.1	3.437	1.570

The effects of dietary ZnONPs supplementation on feed consumption (FC), cumulative feed conversion ratio (FCR) and performance index (PI) are presented in Table 2. Results showed that the amount of feed consumed (FC) per chicks per week was affected by dietary treatments**.** Addition of ZnONPs in broiler diets (T2 and T3) resulted in numerical increased in weekly and cumulative feed consumption than the control (3.257 3.537 and 3.437 for T1, T2 and T3, respectively). Concerning feed conversion ratio (FCR), the data presented in Table 3 indicated that the quantity of feed required per unit of weight was increased in ZnONPs-treated birds as compared with control. The FCR was 1.537 and 1.570 for T2 and T3 respectively compared with 1.625 for T1.

**Table 3:** Effects of ZnONPs supplementation on carcass and internal organs’ relative weight (gm/100 gm) of broiler chicks.

Groups	Carcass	Liver	Provent	Kidney	Heart	Intestine	Cecal	Gizzard
T1	68.29^b^ ± 1.45	1.931^b^ ± 0.125	0.403^a^ ± 0.01	0.639^a^ ± 0.025	0.427^a^ ± 0.014	6.648^a^ ± 0.231	0.831^a^ ± 0.063	2.249^a^ ± 0.147
T2	76.029^a^ ± 0.72	2.152^b^ ± 0.046	0.348^b^ ± 0.013	0.532^b^ ± 0.012	0.403^a^ ± 0.016	4.456^c^ ± 0.275	0.802^a^ ± 0.036	2.018^b^ ± 0.144
T3	72.285^a^ ± 1.07	2.396^a^ ± 0.011	0.36^ab^ ± 0.022	0.546^b^ ± 0.032	0.439^a^ ± 0.021	5.413^b^ ± 0.296	0.806^a^ ± 0.018	2.276^a^ ± 0.102

Values are means ± SEM.

a, b, c—Means in the same column with different superscripts are significantly different (*P*<0.05).

### Carcass characteristics and relative organ weights.

Carcass traits and internal organs weight relative to body weight of broiler chicks, as affected by dietary treatment are tabulated in Table 3. Results showed that supplemented broiler diets with ZnONPs (T2 and T3) caused a significant increase (*P* < 0.05) in carcass yield as compared with control group (T1). Carcass yield was increased at ZnONPs at 40 mg/kg in comparison with other groups. Results also indicated that dietary ZnONPs supplementation (T2 and T3) significantly decreased relative kidney, proventriculus and intestine weights. On the other hand, liver weight was increased at both supplemented levels than control, but was significantly only in T3 chicks with increasing ZnONPs dose. The relative weight of gizzard in T2 birds only was significantly (*P* < 0.05) lower than birds fed on the control one and no significant difference was observed when compared T3 with control chicks, which were statistically similar to each other**.** No significant differences were observed in heart and cecal relative weights between groups**.**

### Blood biochemistry parameters

Table 4 lists the influences of dietary ZnONPs treatments as feed supplement on serum biochemical analysis of broiler chicks. Data of the present research indicated that dietary inclusion of ZnONPs at both levels examined significantly decreased (P < 0.05) serum concentrations of cholesterol (Chol), triglycerides (TG), low density lipoprotein (LDL) and uric acid and increased high density lipoprotein (HDL) value in comparison to control group. As well as, the highest concentration of serum enzymatic activity (ALT and AST) were observed in ZnONPs- treated chicks’ groups (T2 and T3), but was significant only with increase the inclusion level of ZnONPs at 60 mg (T3) as compared with the controls. On the other hand, dietary ZnONPs had insignificant effects on serum glucose and creatinine concentrations.

**Table 4:** Effects of ZnONPs supplementation on serum biochemical parameters of broiler chicks.

Groups	Chol (mg/dl)	HDL (mg/dl)	TG (mg/dl)	LDL (mg/dl)	Glucose (mg/dl)	Uric (mg/dl)	Creat (mg/dl)	ALT (U/L)	AST (U/L)
T1	190.850^a^ ± 3.46	59.771^b^ ± 1.02	146.187^a^ ± 1.99	94.509^a^ ± 2.31	118.892^a^ ± 2.92	6.786^a^ ± 0.32	0.643^a^ ± 0.03	36.863^b^ ± 1.52	26.70^b^ ± 1.07
T2	172.496^b^ ± 1.94	63.120^a^ ± 2.25	139.046^b^ ± 2.29	83.088^b^ ± 1.34	118.438^a^ ± 1.71	6.213^a^ ± 0.27	0.594^a^ ± 0.03	38.205^b^ ± 1.22	27.241^b^ ± 1.58
T3	177.230^b^ ± 2.1	65.0972^a^ ± 1.63	141.892^ab^ ± 1.65	81.890^b^ ± 2.61	117.960^a^ ± 2.26	5.991^b^ ± 0.27	0.578^a^ ± 0.02	42.061^a^ ± 1.43	30.617^a^ ± 1.29

Values are means ± SEM.

a, b—Means in the same column with different superscripts are significantly different (*P* < 0.05).

### Zn content (ppm) in serum and body tissues (liver, breast and thigh muscle)

Significant differences (*P* < 0.05) among the treatments in the Zn content of liver, breast, thigh, and blood serum were observed (Table 5). It could be noticed that supplementation of nano form of Zn into broiler diets at both levels had a dose dependent increase in the concentration of Zn in serum, breast, thigh muscles and liver as compared to those of control birds.

**Table 5:** Effects of ZnONPs supplementation on tissues and serum Zn concentration (ppm) of broiler chicks.

Groups	Zn (serum)	Zn (breast)	Zn (thigh)	Zn (liver)
T1	1.107^c^ ± 0.11	17.92^b^ ± 0.07	21.97^c^ ± 0.11	36.74^c^ ± 0.15
T2	1.563^b^ ± 0.06	20.55^a^ ± 0.18	22.4^b^ ± 0.22	42.4^b^ ± 0.32
T3	2.017^a^ ± 0.09	21.71^a^ ± 0.11	27.61^a^ ± 0.12	49.03^a^ ± 0.21

Values are means ± SEM

a, b, c—Means in the same column with different

Superscripts are significantly different (*P*<0.05)

### Histological observations

Prominent and significant histological changes related to dietary treatments employed in the present study were demonstrated in the liver, kidney and intestinal tissues.

### A-intestine

**Figure 1:**
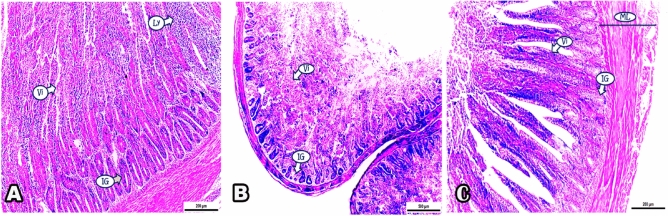
(**A**–**C**) Photomicrographs demonstrating the outcomes of ZnONPs supplement on the intestinal tissue of examined broilers. In 40 mg ZnONPs group, (**A**) Noticeable desquamation of the intestinal villi, increase in cell production of intestinal villi (Vi), intestinal gland (IG), and aggregated lymphocytes (Ly). In 60 mg ZnONPs group, (**B**) Marked degeneration, apoptosis of intestinal villi (Vi), and atrophy of intestinal gland (IG). In control group (**C**) Typical microvilli (Vi), intestinal gland (IG), and intact muscular layer (ML). (H&E staining, A&C:100 × magnification, scale bar = 200 μm, B: 40 × magnification, scale bar = 500 μm).

Photomicrographs presenting the effect of ZnONPs supplement on the intestinal villi (Vi) and intestinal crypt / gland (IG) of tested broilers. Histological examination for the sections of intestine of broiler chicks fed a supplemented diet with 40 mg/kg ZnONPs are shown in Fig. 1A. The results showed obvious desquamation of the intestinal villi, profound increase in cell production of intestinal villi, goblet cells, intestinal crypt depth, and aggregated lymphocytes.

At the same time, histological study for the sections of intestine from the birds group fed supplemented diet with 60 mg ZnONPs, exhibited marked degeneration, apoptosis of intestinal villi, and atrophy of intestinal crypt and no changes recorded in musculature layer (Fig. 1B). On the other hand**,** examined intestine of broiler chicks of control group presenting typical microvilli lined by enterocytes bearing brush border membrane and intestinal crypts and intact muscular layer (Fig. 1C).

Morphometric analysis of intestinal villi length and crypt depth confirmed a statistical difference between T2 group (40 mg ZnONPs) and other groups. In contrast, no statistical difference recorded between T1 (Control group), and T3 (60 mg ZnONPs) (Table 6).

**Table 6:** Effects of ZnONPs supplementation on intestinal villi length and crypt depth of broiler chicks.

Groups	Intestinal villi length	Intestinal crypt depth
T1	8.94^b^ ± 0.46	3.19^b^ ± 0.17
T2	11.41^a^ ± 0.6	5.37^a^ ± 0.59
T3	9.63^b^ ± 0.32	3.45^b^ ± 0.32

Values are means ± SEM.

a, b—Means in the same column with different superscripts are Significantly different (*P*<0.05).

### B-Liver

**Figure 2:**
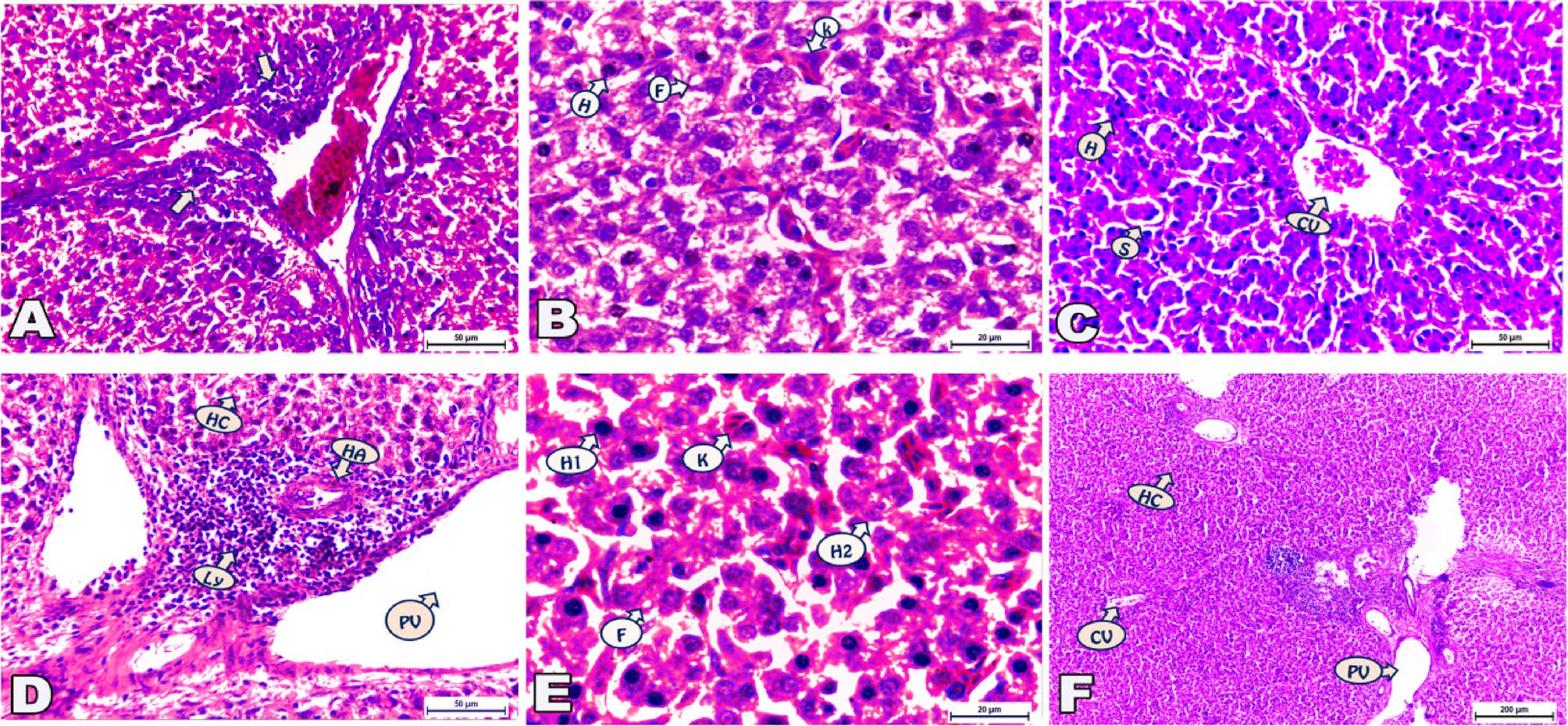
(**A**–**D**) Photomicrographs displaying the effect of ZnONPs supplement on the normal hepatic architecture of the tested broilers. In 40 mg ZnONPs group, (**A**) Inflammatory cells infiltration (arrows) presented in portal area. (**B**) Hepatocytes (H) were dispersed between fat droplets (F), von Kupffer cells (k), and dilated sinusoids. In 60 mg ZnONPs group, (**C**) Area of central vein (CV) supported by hepatocytes (H) and sinusoids (S). (**D**) Portal area displayed portal vein (PV), hepatic artery (HA), inflammatory cells infiltration (Ly), and Hepatic cords (HC). (**E**) Parenchyma of liver tissue marked hepatocyte in normal (H1) and karyorrhexis forms (H2), von Kupffer cells (K) engulfed RBCs besides fat droplets (F), and dilated sinusoids. In Control group (**F**) The liver assembled a normal structure of central vein (CV), portal vein (PV), and hepatic cords (HC). (H&E staining, A&C&D: 400 × magnification, scale bar = 50 μm, B&E: 1000 × magnification, scale bar = 20 μm, F:100 × magnification, scale bar = 200 μm).

Photomicrographs for the section of the liver displaying the effect of ZnONPs supplement on the normal hepatic architecture of the tested broilers are presented in Fig. 2.

Histopathological finding in the sections of the livers of 40 mg ZnONPs—fed broiler revealed loss of hepatic cords arrangement and aggregation of inflammatory cells encircled portal area (Fig. 2A) Moreover, hepatocytes were dispersed between fat droplets, von Kupffer cells, and dilated sinusoids (Fig. 2B). Microscopical examination of the liver of chicks treated with 60 mg ZnONPs in their diets exhibited normal architecture around area of central vein supported by hepatocytes and sinusoids (Fig. 2C).Unlike portal area that highlighted with aggregated inflammatory cells enclosing portal vein, hepatic artery, and hepatic cords (Fig. 2D) Also, parenchyma of liver tissue in this group revealed some normal hepatocytes and others in karyorrhexis form. Von Kupffer cells engulfed RBCs besides fat droplets and dilated sinusoids were also detected (Fig. 2E). on the other hand, histological examination in the sections of the liver of the control group in the present study revealed that no histopathological alteration or any appreciable changes were observed. The liver assembled a normal structure of central vein, portal area and hepatic cords (Fig. 2F).

### C-Kidney

**Figure 3:**
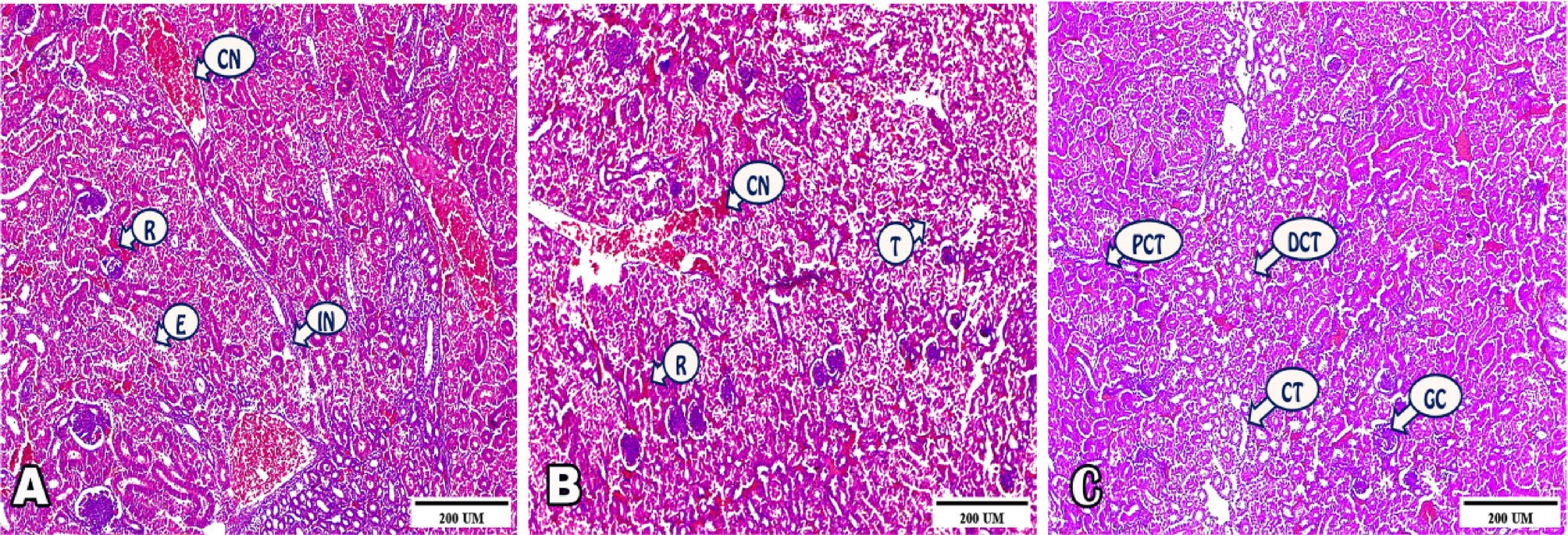
(**A**–**C**) Photomicrographs revealing the outcome of ZnONPs supplement on renal cortex structure of the examined broilers. (**A**) Section in 40 mg ZnONPs group exposed congested vessels (CN), extravasation of blood cells between renal tubules (R), inflammatory cells infiltration (IN), and exudate inside some tubules (E). (**B**) Section in 60 mg ZnONPs group highlighted degeneration of renal tubules (T), extravasated RBCS (R), and congestion in blood vessels (CN). (**C**) Section in negative group demonstrated normal structure of renal cortex; glomerular corpuscle (GC), proximal convoluted tubules (PCT), distal convoluted tubules (DCT), and collecting tubules (CT). (H&E staining, 100 × magnification, scale bar = 200 μm).

Photomicrographs of renal cortex represented the effect of ZnONPs supplementation as following; 40 mg ZnONPs group displayed congested blood vessels, extravasation of blood cells between renal tubules and surrounding glomerular corpuscle, inflammatory cells infiltration, and epithelial desquamation and exudate noticed inside some tubules (Fig. 3A). Conversely, 60 mg ZnONPs group highlighted degeneration of renal tissue including tubular disorganization and apoptotic cells, extravasated RBCS throughout tissue, and congestion in blood vasculature (Fig. 3B). Approaching control group, normal renal cortex structure emphasized with glomerular corpuscle, proximal convoluted tubules, distal convoluted tubules, and collecting tubules (Fig. 3C).

## Discussion

### Productive performance

The findings of the present study confirm that supplementing basal diet with ZnONPs to broiler chicks resulted in a significant increase in body weight, increased feed consumption and improved feed conversion ratio compared with control chicks. In agreement with our results, Nano ZnO at 2.5 ppm significantly increased body weight gain, feed intake, and improved feed conversion ratio^27^. Also, the same observations were recored with 0.2–0.3 g/kg of ZnONPs^28^. Broiler chicks fed with ZnONPs at 90 mg/kg for 35 days of age showed an improvement in body weight compared with the bulk zinc oxide-administered group at 3000 mg/kg diet^29^. In addition, previous results in the literature confirmed that ZnONPs at 20 to 60 mg/kg of diet could be appropriate levels and a considerable source of Zn to enhance BWG and achieve a better FCR of broiler chickens^30,31^ and this was matched with the present work and that 40 mg/kg Nano-ZnO is the optimal level in diets.

The performance improvement may be attributed to the unique nature of ZnONPs that increased the intestinal absorptive capacity by increasing villi length and width, mucosal length, and crypt depth^32^. As such, the higher absorption efficiencies of ZnONPs resulting in the improved bioavailability of Zn^33^.

The positive effect of ZnONPs on growth performance through the current study is corroborated with earlier works reported and could be attributed to the fact that Zn plays an important role in the overall physiological and performance of poultry, as it is the integral component of more than 300 enzyme systems known as metalloenzymes^34^, which are involved in metabolism, nucleic acids, lipids, and protein^35^. Furthermore, Zn deficiency in animals is characterized by appetite reduction, decreased FI, decreased hepatic production of insulin growth factor, circulating levels of growth hormone, and growth falling in broilers^36^. It was also suggested that ZnONPs increased sucrase concentration in the small intestine thereby increasing carbohydrate absorption^36^, antioxidant, and anti-stress properties, and affect intestinal microbiome of broiler chickens^37^.

### Carcas*s* and relative organ weights

The finding in the present results showed that ZnONPs at 40 or 60 mg/kg (as a supplement of broiler diets) affected the carcass weight and internal organ weights as a percentage of live body weight. Increasing carcass and liver weight and decreasing kidney, proventriculus, gizzard and intestine weight was observed as compared with control. In agreement with these results, the relative weight of proventriculus at 40 and 100 mg ZnONPs/kg of diet were significantly lower and the relative weight of the intestine was decreased by lowering the ZnONPs levels with significant differences at 40 and 20 mg/kg of diet compared with the control^28^. Moreover, weight of small intestine was lower and the liver weight was significantly increased as percentage of LBW in birds fed diet supplementation with 60 or 90 mg ZnONPs/kg. The recorded increase in liver weight could be attributed to the better Zn bioavailability in the form of nanoparticles which resulted in higher Zn retention in liver after absorption and inters to the blood portal ^38^. Similarly, dietary ZnONPs significantly increased carcass yield and weight, and dressing percentage at a concentration range from 40 to 90 ppm^15^.

### Blood biochemistry parameters

Here, the blood parameters of the ZnONPs based diet groups varied significantly in terms of Chol, HDL, TG, LDL, ALT, AST, uric acid and serum creatinine concentration. Mahmoud and co-workers clarified that dietary inclusion of ZnONPs at 20 ppm resulted in a significant reduction in uric acid concentration and serum TG, while serum HDL level was significantly higher as compared with control broiler group^30^. Also, Nano Zn at levels of 0.2 and 0.3 g/kg diet significantly decreased total cholesterol and LDL levels and had the highest value for HDL in the serum as compared to control in growing Japanese quail diets^28^. In similar direction, serum total cholesterol and TG decreased significantly and activities of liver enzymes (AST and ALT) remained unchanged in the serum of Japanese quail fed ZnONPs at 30 or 60 mg/kg compared to the control^39^. Current results also came in accordance with Attia and co-workers who recorded a reduction in cholesterol, TG, creatinine, uric acid and increased HDL with the supplementation of ZnONPs at level of 20 or 40 mg/kg diet^40^. Moreover, these observations were attributed to the ability of Zn to induce glucagon secretion and suppress insulin secretion^41^.

The significant increase in serum HDL and reduced blood serum TG and cholesterol in the present study is parallel with two previous studies^15^ on laying hen and broiler respectively administrated ZnONPs. Previous studies attributed thse observations to the improvement in calories and fat intake after Zn supplementation^42^. Besides, the important role of Zn in enzymes systems where it forms an integral part of several metalloenzymes which plays a vital role in fat metabolism responsible for lipid digestion and absorption^43^. Meanwhile, the decrease in cholesterol could be attributed to the preventive role of Zn to cholesterol from absorption in gastro intestinal tract and may promote the growth and activity of lactic acid bacteria, which reduces the cholesterol level^44^.

### Tissue zinc concentrations

Minerals in the nano form showed better ability to pass through the small intestine and distribute in the body than their inorganic and organic minerals^45^. In the current study, significant differences among treatments in the Zn concentration of liver, serum, breast, and thigh muscles. Supplementation of ZnONPs at both doses increased the amount of Zn in serum and all tissues examined compared with the control group and these increases linearly with increasing dietary ZnONPs levels. These results came in agreement with those reported for broilers^29^ and layers^33^ where, the highest levels of zinc were detected in the livers, serum and muscles of birds supplemented with ZnONPs when compared with the control one. Additionally, El-Bahr and co-workers observed that inclusion of ZnONPs in the diet induced a significant increase in the Zn concentration in liver of Japanese quails compared to the control^39^. Similar results were obtained in rabbit supplemented by ZnO at concentration 30 and 60 mg/kg with a significantly increased hepatic and serum zinc concentrations, which partly supported better absorption of ZnO and subsequently the positive relationship between ZnO supplementation and growth performance of rabbits^46^. Gundogdu and co-workers concluded that Zn in the nano form is able to penetrate into the hepatic cells via blood or interstitial space and being the liver as the main organ of Zn metabolism^47^. Besides, the dietary Zn supplementation linearly increases plasma and liver Zn concentrations in broilers^9^. It is proved that the liver is sensitive to Zn supplementation because this organ acts as a Zn reservoir in the body, thus, it is expected that the birds supplemented with Zn should have a higher content of this element in their liver^48^. In this respect, our results received support from the earlier work, where Zn content in chicken thigh were higher than in breast^49^. The concentration of Zn differes in tissues due to the morphology, biochemical and functional state of the tissues. Moreover, Zn deposition was significantly increased in breast muscle and liver with dietary substitution of nano Zn than inorganic one^35^.

### Histological section

#### A-Intestine

The impact of ZnONPs supplement on the intestinal villi and intestinal crypt of tested broilers was revealed. Histological examination for the sections of intestine of broiler chicks fed a supplemented diet with 40 mg/kg ZnONPs exhibited obvious desquamation of the intestinal villi, profound increase in cell production of intestinal villi and intestinal crypt, and aggregated lymphocytes. This was in consistent with the data recorded for the dietary replacement of 60 mg inorganic zinc oxide with 45 and 30 mg of nano zinc/kg diet which, significantly increased villi length and width, crypt depth and villi length/crypt depth ratio compared with broiler chicks group fed on the basal diet, which indicate the improvement of absorptive capacity of different nutrients and improved feed efficiency^15^. Similarly, with the pervious results when chicks fed diets with supplemental ZnONPs at 50 ppm^50^. Also, supplementation of 40 mg ZnONPs was a considerable feed additive for poultry with beneficial effects on intestinal changes including increase in villus surface area, highet and total goblet cell count^51^. The authors attributed the higher villus height to higher bioavailability of ZnONPs, so maintaining epithelial barrier integrity and function.

The increase in crypt depth of chicken supplemented with different levels of ZNONPs might provide more surface area for nutrient absorption by increasing enterocyte proliferation and intestinal mucin secretion^52^. Also, it was indicated that zinc repaired intestinal injury by reducing the apoptotic index of ileal epithelial cells, enhancing villus height and crypt depth^53^. This can clarify the results of this study in the sections of intestine from the birds group fed supplemented diet with 60 mg ZnONPs, they suffered from marked tissue degeneration, apoptosis of intestinal villi, and atrophy of intestinal crypt.

#### B-Liver

Present results were in line with the effect of high (900 mg/kg b.w.), intermediate (600 mg/kg b.w.) and low (300 mg/kg b.w) concentrations of Zn on histology of the liver of broiler chicks^29^. Previous literature displayed that ZnONPs could raise neutrophils numbers with attraction to some proteins such as immunoglobulin and lipoproteins^54^. These pathological changes and their toxic effects may be attributed to their solubility, resulting in increased intracellular Zn^55^. Moreover, nanoparticles are expected to increase the existence of inflammation in diverse organs, consequently leading to the spreads of the inflammatory reactions^56^. Watson and co-workers added that the administration of ZnONPs hindered Kupffer cell phagosomes motility and thus conducted the hepatic injury^57^.

#### C-Kidney

Supplementation 40 mg ZnONPs to broilers displayed congested blood vessels, extravasation of blood cells between renal tubules and surrounding glomerular corpuscle, inflammatory cells infiltration, and epithelial desquamation and exudate noticed inside some tubules**.** Moreover, 60 mg ZnONPs group highlighted degeneration of renal tissue including tubular disorganization and apoptotic cells, extravasated RBCS throughout tissue, and congestion in blood vasculature. This agreed with previous work where ZnONPs exerted a destructive effect on the kidney^58^. Besides, these ZnONPs had a toxic effect on the renal tissue at a high concentration. Furthermore, kidney tissue of animals treated with ZnONPs showed necrosis and apoptosis^59^. The cytotoxixity and DNA damage present in the kidney cells were attributed to oxidative stress after ZnONPs treatment that increased MDA contents and decreased SOD and GPx enzymes activity^60^.

## Conclusion

Generally, under the condition of this study, the obtained results confirmed that dietary supplementation of the biologically synthesized ZnONPs by the endophytic fungud *Alternaria tenuissima* at inclusion level of 40 or 60 mg/kg of diet have significant positive effects in improvements of broiler performance, physiological status, carcass traits, serum biochemical parameters, lipid profile, intestinal health, concentration of Zn in serum and tissue, and finally health status of broiler chicks. current results showed also that the delivery of Zn in the forms of ZnONPs at concentration of 40 mg/kg to broiler diets was more efficient, beneficially and could be used as safe dose without any detrimental effect than high levels.

## Data avialabilty

All data generated or analyzed during this study are included in this published article.

## Abbreviations

ZnONPs: Zinc oxide nanoparticles
CP: Crude protein
ME: Metabolizable energy
Chol: Cholesterol
TG: Triglycerides
LDL: Low density lipoprotein
HDL: High density lipoprotein
ALT: Alanine aminotransferase
AST: Aspartate aminotransferase
CREAT: Creatinine
